# A novel miRNA-based classification model of risks and stages for clear cell renal cell carcinoma patients

**DOI:** 10.1186/s12859-021-04189-2

**Published:** 2021-05-25

**Authors:** Eskezeia Y. Dessie, Jeffrey J. P. Tsai, Jan-Gowth Chang, Ka-Lok Ng

**Affiliations:** 1 Department of Bioinformatics and Medical Engineering, Asia University, Taichung, Taiwan; 2 Department of Laboratory Medicine, China Medical University, Taichung, Taiwan; 3 Department of Medical Research, China Medical University Hospital, China Medical University, Taichung, Taiwan; 4 Center for Artificial Intelligence and Precision Medicine Research, Asia University, Taichung, Taiwan

**Keywords:** Clear cell renal cell carcinoma, Biomarkers, MicroRNAs, Survival analysis, Regularized cox model, Machine learning classifiers

## Abstract

**Background:**

Clear cell renal cell carcinoma (ccRCC) is the most common subtype of renal carcinoma and patients at advanced stage showed poor survival rate. Despite microRNAs (miRNAs) are used as potential biomarkers in many cancers, miRNA biomarkers for predicting the tumor stage of ccRCC are still limitedly identified. Therefore, we proposed a new integrated machine learning (ML) strategy to identify a novel miRNA signature related to tumor stage and prognosis of ccRCC patients using miRNA expression profiles. A multivariate Cox regression model with three hybrid penalties including Least absolute shrinkage and selection operator (Lasso), Adaptive lasso and Elastic net algorithms was used to screen relevant prognostic related miRNAs. The best subset regression (BSR) model was used to identify optimal prognostic model. Five ML algorithms were used to develop stage classification models. The biological significance of the miRNA signature was analyzed by utilizing DIANA-mirPath.

**Results:**

A four-miRNA signature associated with survival was identified and the expression of this signature was strongly correlated with high risk patients. The high risk patients had unfavorable overall survival compared with the low risk group (HR = 4.523, *P*-value = 2.86e−08). Univariate and multivariate analyses confirmed independent and translational value of this predictive model. A combined ML algorithm identified six miRNA signatures for cancer staging prediction. After using the data balancing algorithm SMOTE, the Support Vector Machine (SVM) algorithm achieved the best classification performance (accuracy = 0.923, sensitivity = 0.927, specificity = 0.919, MCC = 0.843) when compared with other classifiers. Furthermore, enrichment analysis indicated that the identified miRNA signature involved in cancer-associated pathways.

**Conclusions:**

A novel miRNA classification model using the identified prognostic and tumor stage associated miRNA signature will be useful for risk and stage stratification for clinical practice, and the identified miRNA signature can provide promising insight to understand the progression mechanism of ccRCC.

**Supplementary Information:**

The online version contains supplementary material available at 10.1186/s12859-021-04189-2.

## Background

Renal cell carcinoma (RCC) is one of the top ten cancer diagnoses, and it accounts for 3–5% of all new cases in females and males [1]. Recently, there are more than 140,000 death per year associated with RCC [2]. Clear cell renal cell carcinoma (ccRCC) is the most common RCC subtype and it represents 70–80%, of all renal malignant tumors [3]. Despite many advances in effective therapeutic and diagnostic strategies in ccRCC, and the overall survival rate is still poor, particularly for advanced-stage ccRCC patients[4]. CcRCC has poor prognosis due to the resistance to chemotherapy and radiotherapy[5]. Late tumor staging is the main risk factor of ccRCC patients [6] and detection of ccRCC patients at early-stage is crucial for better diagnosis and treatment options.

Currently, the development of next generation sequencing (NGS) technology has enabled researchers to explore genetic alterations in tumorigenesis and discovering molecular biomarkers for many cancers[7]. NGS allows to examine the possible contributions of the upstream molecular regulators of gene expression such as miRNAs. MiRNA play critical roles in regulating various physiological and pathological processes, including regulation of cell division, apoptosis, cell maturation, angiogenesis, metastasis, migration, invasion, differentiation of cells, metabolism, and proliferation by negative regulation of gene expression [8–10]. Moreover, in various cancers, dysregulated miRNAs can be used as biomarkers [11–13]

Statistical and machine learning approaches have been used to predict gene sets as biomarkers for patients with ccRCC [14]. Ng and Taguchi employed the tensor decomposition method to identify miRNA signature in ccRCC [15]. Previously, studies were used miRNA expression profiles of liver and breast cancer patients, followed by a support vector machine (SVM) with genetic algorithm, to predict the early and advanced stages [16, 17]. Recently, miRNA profiles were used to detect lung cancer subtypes [18]. Several studies have reported miRNA biomarkers in ccRCC. For example, a three-miRNA signature including miR-21, miR-155 and miR-584 is associated with survival in ccRCC [11]. Zhao and Bai identified 13-miRNA signature associated with overall survival in ccRCC [19]. However, the study of multi-miRNA signature models to predict the risks and tumor stages of ccRCC patients are still limited and hence, we aimed to select a small set of miRNAs as signature that can predict risk as well as tumor stages in ccRCC patients using genomic profiles, so that identified miRNA signature can provide promising insight to understand the progression and development mechanism of ccRCC.

In this study, we proposed a computational method for identifying prognostic-associated miRNA signature as well as predicting the early and late tumor stages of ccRCC using miRNA expression profiles. We identified a four miRNA signature associated with the prognosis of ccRCC from high-dimensional miRNA expression profiles using multivariate Cox regression with Elastic-net, Lasso and Adaptive lasso penalties followed by best subset regression analysis. The prognostic risk model involving four miRNA signature effectively classified ccRCC patients into high and low risk groups; prognosis was significantly worse in high-risk group when compared with low-risk groups. Furthermore, we extracted significant miRNAs that can distinguish early and late tumor stages using various machine learning approaches. We identified a six miRNA signature strongly related to tumor stages of ccRCC patients. The five machine learning algorithms were used to evaluate classification performance of a six miRNA signature using independent testing set. Finally, the SVM algorithm achieved the best classification performance when compared with other classifiers.

## Results

We proposed a computational method including penalized Cox models and machine learning approach to identify miRNA signature for risk and tumor stage prediction using miRNA profiles, which consists of several steps as described in detail in the “Methods” section. To develop optimal prognostic predictive model for ccRCC patients, combined penalized Cox models (including Elastic-net, Lasso and Adaptive lasso), best subset regression and risk score model were used. Furthermore, a combined machine learning approach was used to prioritize and identify miRNA signatures associated with early and late tumor stages in ccRCC patients. The systematic pipeline of the overall process is shown in Additional file 1: Fig. S1.

### Identification of dysregulated miRNAs in ccRCC patients

After TCGA-ccRCC data quality assessment, preprocessing and normalization, a total of 1046 miRNA expression profiles were used for differentially analysis based on “limma” package in R. We identified 124 differentially expressed miRNAs (DEMs), of which 80 downregulated and 44 upregulated miRNAs in 254 primary tumor tissue samples compared with 71 normal samples, using the criteria of absolute value log2foldchange > 1 and Benjamin-Hochberg (BH) adjusted *P*-value < 0.05 (Additional file 1: Table S1). These abnormal miRNAs were used for subsequent survival and stage classification model development.

### Identification of prognostic-associated miRNAs and development of risk classification model

**Fig. 1:**
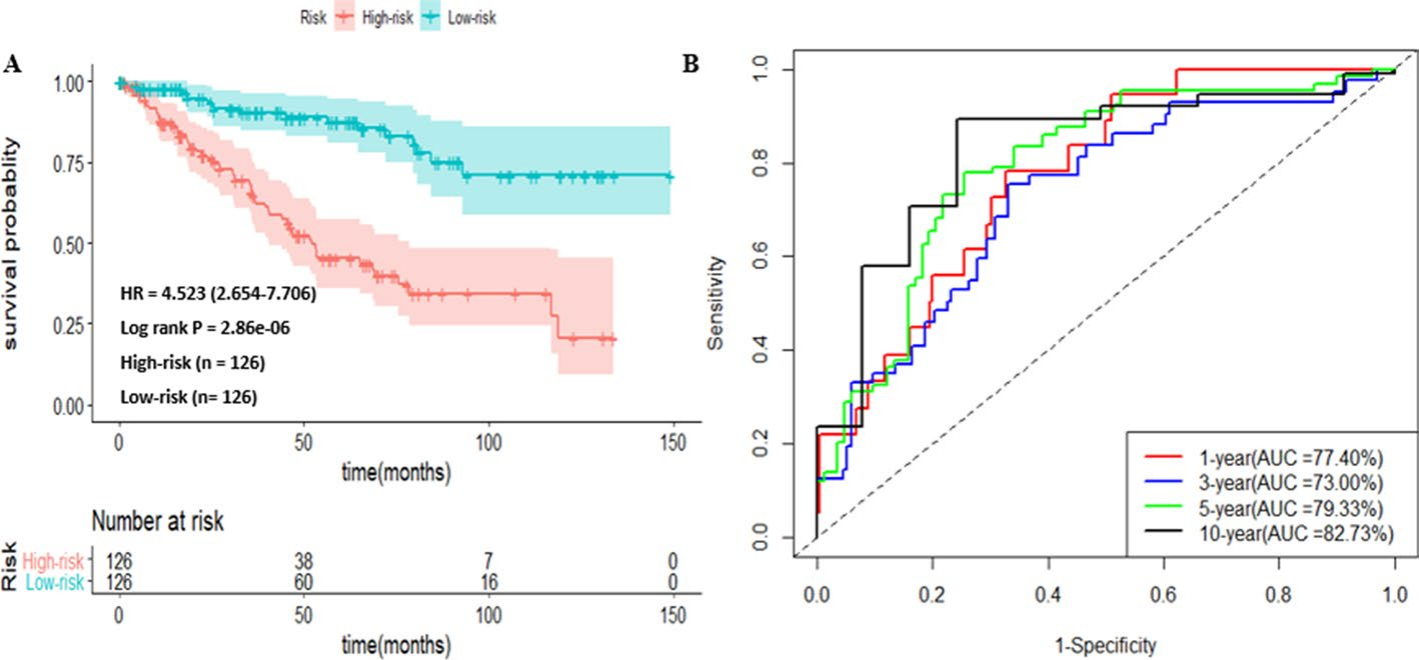
Prognostic classification of ccRCC patients using the risk score model. **a** Kaplan–Meier (KM) survival analysis for high risk and low risk subgroup stratification based on the risk score model. **b** Validation of risk score classification performance by time dependent ROC analysis

DEMs that altered in tumor samples are potential prognostic and diagnostic signatures. To identify significant prognostic DEMs, the TCGA cohort of ccRCC patient (n = 252) having survival information were used. Subsequently, three regularized survival methods (including Elastic-net, Lasso, and Adaptive lasso) with ten-fold cross validation were implemented to obtain the optimal lambda (λ) values that obtained from the smallest partial likelihood deviances. The estimated optimal penalty parameter λ values for the three algorithms were $\lambda_{opt}^{Enet}$ = 0.093, $\lambda_{opt}^{Lasso}$ = 0.056 and $\lambda_{opt}^{Ad.lasso}$ = 0.015 and these optimal tuning parameters were used to choose informative features (miRNAs) that were associated with patient survival. Elastic-net, Lasso, and Adaptive lasso algorithms identified 13 miRNAs, 11 miRNAs and 6 miRNAs respectively (Table 1). A union of candidate miRNAs selected by the three methods including: miR-21, miR-223, miR-146b, miR-30b, miR-3613, miR-187, miR-203, miR-514-3, miR-129-2, miR-200a, miR-508, miR-1.2 and miR-934 were used for BSR analysis. Then, all subset prognostic models created by the identified 13 miRNAs were assessed using BSR analysis based on the  “glmulti” package in *R* and finally we obtained an optimal miRNA prognostic model with four-miRNA signatures (including miR-30b, miR-21, miR-187 and miR-150 200a) having the smallest AIC value (Additional file 1: Fig. S2). Then, using the regression coefficient obtained from the result of multivariate Cox regression analysis of the four-miRNA signatures (Additional file 1: Table S2), we developed a risk score (*RS*) model, which is given by, *RS* = (0.525 $\times$ miR-30b expression) + (1.485 × miR-21 expression) + (0.485 $\times$ miR-187 expression) − (0.320 $\times$ miR-200a expression). Based on this risk score definition, we stratified ccRCC patients into high risk and low risk groups. The Kaplan–Meier curve shows that high risk group related to poor overall survival time relative to low risk group (Fig. 1a). Furthermore, the AUC values of the time dependent ROC curve were 77.40%, 73.00%, 79.33% and 82.73% for a 1-year, 3-year, 5-year and 10-year survival (Fig. 1b). These results demonstrated that the time dependent risk prediction based on a four-miRNA signature can be used for risk assessment for ccRCC patients.

**Table 1:** The penalized Cox regression coefficients of selected miRNAs from three methods

Gene name	Elastic-net	Lasso	Adaptive lasso
miR-21	0.513	0.705	1.049
miR-223	0.117	0.129	0.117
miR-146b	0.083	0.017	–
miR-30b	0.068	0.057	0.061
miR-3613	0.058	0.034	–
miR-187	0.045	0.053	–
miR-203	− 0.011	–	–
miR-514-3	− 0.019	–	–
miR-129-2	− 0.037	− 0.003	–
miR-200a	− 0.071	− 0.060	–
miR-508	− 0.130	− 0.151	− 0.349
miR-1.2	− 0.146	− 0.171	− 0.271
miR-934	− 0.268	− 0.205	− 1.248

### Univariate and multivariate analysis for risk prediction evaluation

In order to further examine whether the identified miRNA signature can be used as independent predictor, we performed univariate and multivariate Cox analysis (Table 2). The results of univariate Cox analysis indicated that age, pathological stages and the miRNA signature are significantly correlated with overall survival. Meanwhile, multivariate analysis showed that pathological stage (*P*-value = 0.048), pathological M stage (*P*-value = 0.035) and four miRNA signature (*P*-value = 4.23e−05) are significantly associated with unfavorable prognosis.

**Table 2:** Univariate and multivariate Cox analyses showed risk score is an independent risk factor

variables	Univariate Cox analysis		Multivariate Cox analysis	
	*P*-value	HR (95% CI)	*P*-value	HR (95% CI)
Age	0.001*	1.808 (1.324–2.47)	0.061	1.598 (0.978–2.610)
Gender	0.757	0.953 (0.702–1.294)	0.078	0.610 (0.352–1.058)
Pathological stage	2e−16*	3.912 (2.857–5.362)	0.048*	3.303 (1.028–10.949)
Pathological T stage	3.8e−16*	3.204 (2.369–4.331)	0.272	0.573 (0.212–1.546)
Pathological M stage	2e−16*	4.254 (3.117–5.805)	0.035*	1.957 (1.048–3.655)
Pathological Grade	3.42e−08*	2.598 (1.851–3.646)	0.217	1.462 (0.804–2.633)
Four-miRNA signature	2.14e−08*	4.628 (2.707–7.911)	4.23e−05*	4.324 (2.385–7.839)

### Identification of miRNA signature for classification of tumor stages

**Fig. 2:**
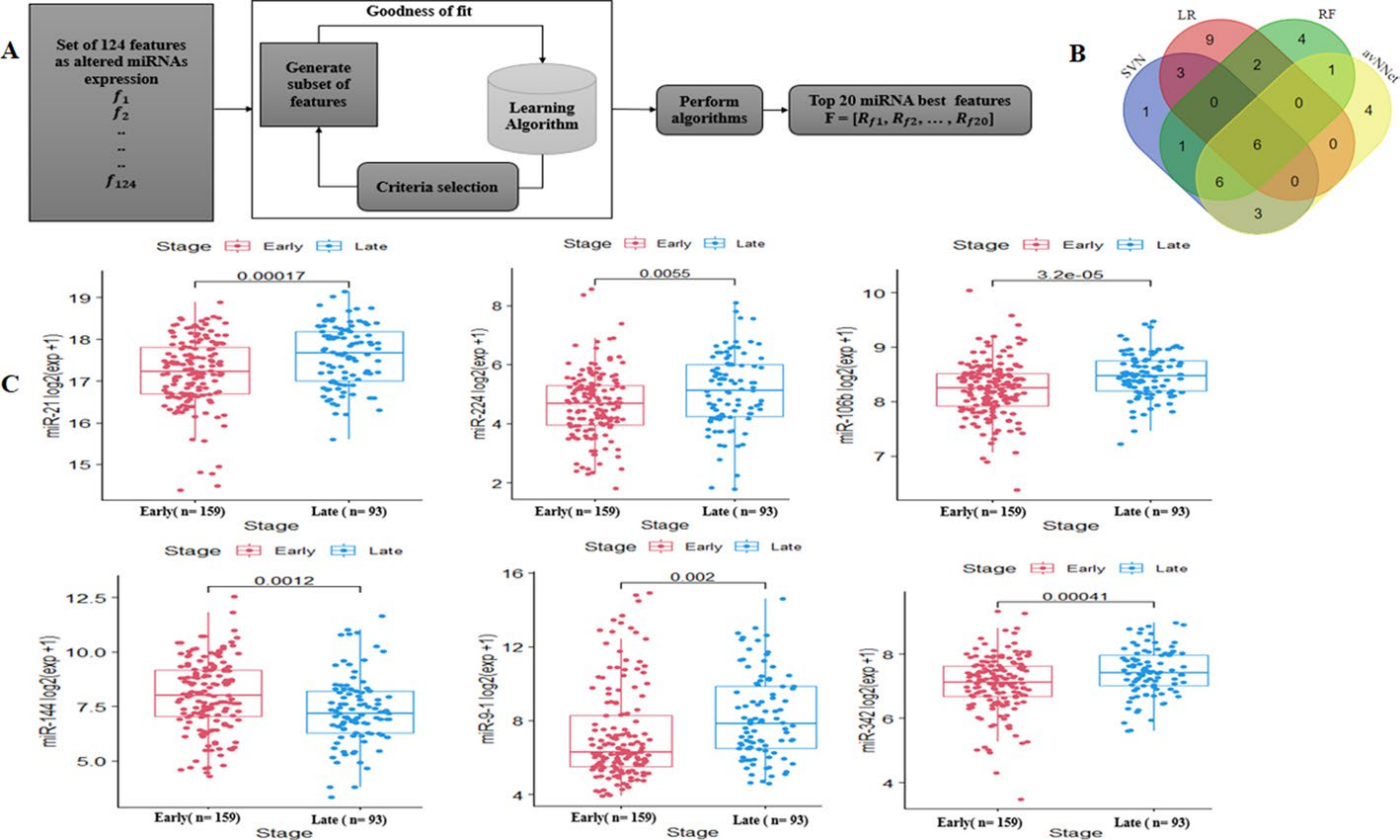
Stage associated features (miRNAs). **a** Implementation of feature selection using various machine learning algorithms. **b** Common features among top 20 ranked miRNAs selected by four ML learning algorithms. **c** Results of six differentially expressed miRNAs between early and late stage of ccRCC patients

In order to identify tumor stage associated miRNA signatures, 124 DEMs expression profiles and the corresponding ccRCC patients with stage information (n = 252) were used. We applied ensemble learning feature selection algorithms including logistic regression (LR), random forest (RF), support vector machine with radial kernel (SVMR) and average neural network (avNNet) model to identify optimal classifier of tumor stages. The maximum Relevance Minimum Redundancy (mRMR) algorithms were used to identify miRNA features having the most correlation with tumor stage and the least correlation with miRNA features themselves. The detail of mRMR algorithm is described in Fig. 2a. The utility of mRMR based ensemble ML algorithm is enhancing informative feature selection by minimizing the bias that might be introduced by single algorithm. We identified top ranked features of 20 miRNAs using four ML methods and the ranking of miRNAs and their corresponding features importance relevance are shown (Additional file 1: Table S3). To determine the common  number of miRNA features for tumor stage classification, we performed an overlapping analysis of selected miRNAs by these algorithms and six common miRNAs including miR-106b, miR-144, miR-224, miR-9-1, miR-21, miR-342 (Fig. 2b) were identified. These identified miRNAs were used to develop a stage prediction model. We also further verified that the identified 6 miRNA features were differentially expressed between early and late stage groups. The box-plot shows for each miRNA, there was a significant expression difference in early and late stage subgroups (Fig. 2c). Interestingly, the identified 6 stage-associated miRNAs are significantly associated with the overall survival of patients with ccRCC (Additional file 1: Fig. S3).

### SMOTE sampling data and stage classification  performance

In this study, SMOTE sampling was used to solve the class imbalance problem. Balanced dataset obtained from the SMOTE algorithm was then divided into training set and test set (80:20 ratio). The identified six miRNA signature was used to build prediction model based on five ML algorithms—SVMR, LR, Naïve Bayes, avNNet and KNN. The classification performance evaluated after data balancing is shown in Table 3. The accuracy of all models were found to be in the range of 0.62–0.923 with maximum accuracy of 0.923 for SVMR. The highest sensitivity and specificity, MCC and precision were observed for SVMR. To check whether SMOTE data sampling resulted improvement prediction, we used the original data (without SMOTE balancing) and constructed the training set and test set in a 80:20 ratio. Stage prediction and their classification performance delivered by the five ML methods were explored. The best accuracy and sensitively was observed in Naïve Bayes with an accuracy 0.775 and sensitivity 0.778. The details of model performance comparison are shown in (Additional file 1: Table S4). It is noteworthy that SMOTE data balancing algorithm can improve the prediction accuracy of all ML models except Naïve Bayes. The overall model prediction results indicated that the identified six-signature miRNAs can classify tumor stages of ccRCC patients with reasonable prediction performance using miRNAs expression profiles.

**Table 3:** The performance of ML based models constructed by identified six miRNA signatures on balanced training set and test set using the SMOTE algorithm

Algorithms	Methods	Performance measures					
		ACC	Sensitivity	Specificity	MCC	F-score	Precision
SVMR	tenfold	0.990	0.987	0.993	0.981	0.989	0.991
	Test	0.923	0.927	0.919	0.843	0.911	0.895
LR	tenfold	0.688	0.649	0.713	0.357	0.620	0.594
	Test	0.612	0.473	0.716	0.194	0.510	0.553
Naïve Bayes	tenfold	0.761	0.737	0.776	0.508	0.711	0.688
	Test	0.721	0.709	0.730	0.436	0.684	0.661
avNNet	tenfold	0.902	0.918	0.892	0.801	0.882	0.848
	Test	0.783	0.709	0.838	0.553	0.736	0.453
KNN	tenfold	0.889	0.874	0.900	0.773	0.870	0.866
	Test	0.775	0.782	0.770	0.547	0.748	0.717

### Enrichment analysis and biological roles of identified miRNA signatures

The identified miRNA signatures are linked with the development and progression of various cancer types. Overexpression of miR-21 leads to amplified cell proliferation and reduced apoptosis [20] and high expression of miR-21 associated with poor survival in lung cancer and ccRCC [11, 21]. Downregulated miR-30b-5p act as a tumor suppressor to regulate renal cell carcinoma in cell proliferation, metastasis and epithelial-to-mesenchymal transition by targeting G-protein subunit α-13 [22]. Decreased miR-187 in clear cell renal cell carcinoma inhibits cell growth, migration though targeting B7-H3 and correlated with lower survival [23]. MiR-106p-5p upregulation targets several negative regulators of the Wnt/β-catenin pathway [24]. MiR-144 promotes RCC development by hampering mTOR expression [25]. Fujii N et al. reported that higher expression of miR-224 associated with poor progression-free survival and overall survival in ccRCC [26]. MiR-200a regulates epithelial to mesenchymal transition-associated with gene expression and regulates prognosis in colorectal cancer [27]. In addition, miR-200a consistently decreased in RCC and serve as diagnostic biomarker for the early detection of RCC [28].

**Fig. 3:**
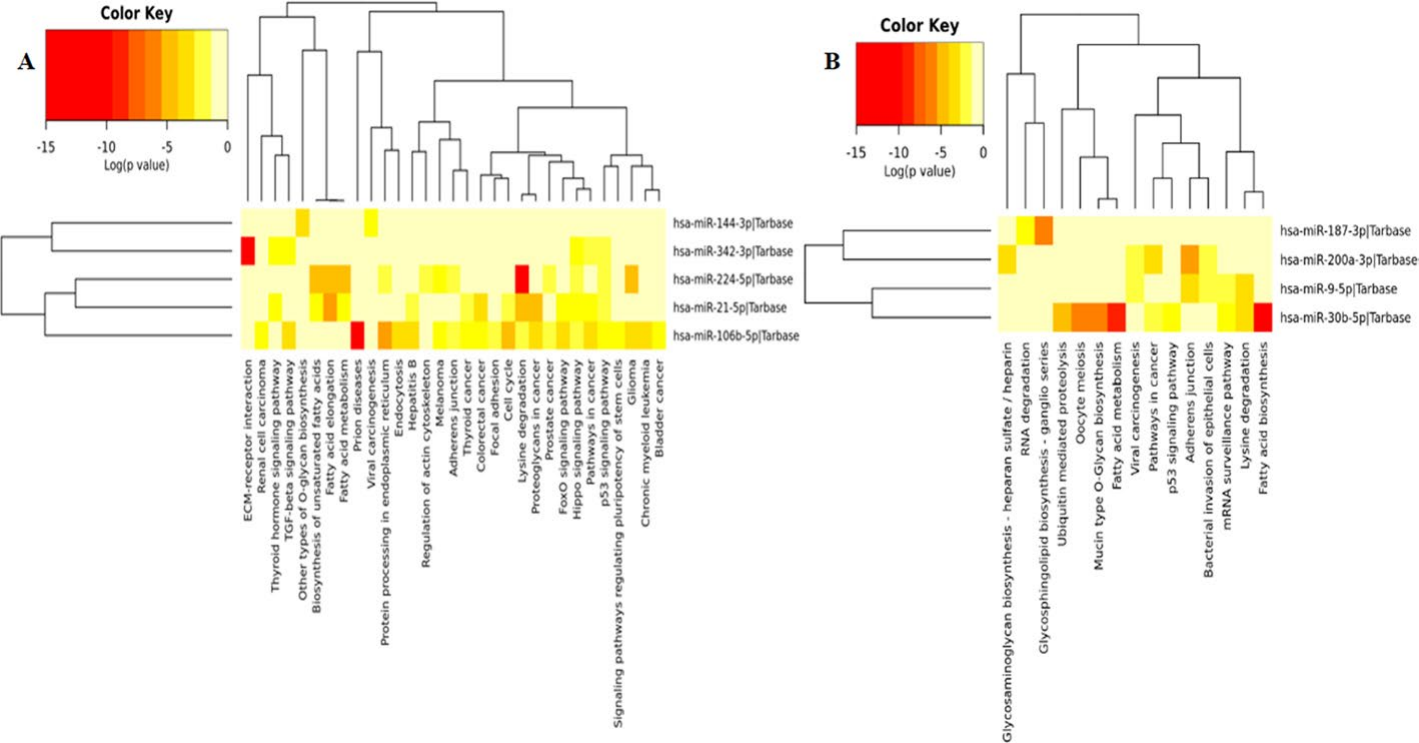
KEGG pathway analysis for up and down regulated miRNA signatures. **a** Enriched KEGG pathways for upregulated miRNA signatures. **b** Enriched KEGG pathways for downregulated miRNA signatures

Biological roles of the identified miRNAs were assessed by using KEEG pathways and GO annotation analyses via DIANA-mirPath. The enriched biological pathways of the upregulated and downregulated identified miRNA signatures are presented in Fig. 3a, b. The upregulated miRNA signatures are enriched in Prion diseases, Lysine degradation, ECM-receptor interaction, Proteoglycans in cancer, Fatty acid elongation,Pathways in cancer, Cell cycle, FoxO signaling pathway, p53 signaling pathway, TGF-beta signaling pathway, Biosynthesis of unsaturated fatty acids, Viral carcinogenesis, signaling pathways regulating pluripotency of stem cells, Renal cell carcinoma and other biological pathways. The detail biological pathways and number of target genes for upregulated miRNA signatures are described in Additional file 1: Table S5. Similarly, downregulated miRNA signatures involved in Fatty acid biosynthesis, Adherens junction, Fatty acid metabolism, Lysine degradation, Pathways in cancer, Viral carcinogenesis, p53 signaling pathway, mRNA surveillance pathway, RNA degradation other KEGG pathways. The detail summary of downregulated miRNA signatures enriched pathways and number of target genes are indicated in Additional file 1: Table S6.

GO analysis also showed that upregulated miRNA signatures participated in cellular nitrogen compound metabolic process, biosynthetic process, mitotic cell cycle, cell death, DNA metabolic process, innate immune response, cell cycle, cell proliferation and others. The detail biological processes and target genes of upregulated miRNA signatures are shown in Additional file 1: Table S7. Furthermore, the biological processes  of downregulated miRNAs are enriched in nucleobase-containing compound catabolic process, macromolecular complex assembly, mitotic cell cycle, cellular protein modification process, catabolic process, biosynthetic process, gene expression, viral process, cellular component assembly, cellular protein metabolic process, small molecule metabolic process, Fc-epsilon receptor signaling pathway, response to stress, cell death and other biological process. The detail biological processes and target genes for downregulated miRNA signatures are shown in Additional file 1: Table S8.

## Discussion

In this study, we showed an efficient strategy to identify miRNA signatures that can stratify high risk and low risk patients as well as classify early and late tumor stages. Several computational and machine leering algorithms have been developed to explore miRNA-associated diseases [17, 29]. Previous studies also used miRNA profiles to identify biomarkers for risk stratification [11, 30]; however, only a few research works have been conducted to explore miRNA signatures for early tumor stage of ccRCC. Therefore, in our study, we proposed a novel hybrid ML strategy to identify miRNA signatures associated with overall survival and tumor stages classification for ccRCC patients. The major findings and contributions of our work are outlined as follow. Identification of risk and stage predictive miRNA signatures with good predictive performance. The use of multivariate Cox regression with Elastic-net, Lasso and adaptive Lasso penalties followed by optimal subset prognostic model selection strategy identified a four novel-miRNA signature, namely miR-30b, miR-21, miR-187 and miR-200a. This signature can effectively stratify high risk and low risk subgroups with good classification performance (time dependent ROC); hence, the four-miRNA signature may be use as a prognostic biomarker. Furthermore, the proposed combined ML algorithms identified six top ranked miRNAs (miR-106b, miR-144, miR-224, miR-9-1, miR-21 and miR-342) based on their relative importance and their classification performance  were evaluated by five ML methods. The classification performance of the six-stage associated miRNAs revealed effective stratifying ability early stage and late stage. The stage classification performance can be further improved if we applied SMOTE algorithm to prepare balanced early and late tumor stage data. The consistency and validation of the predictive models were assessed using an independent test set. The biological significance of the identified miRNA signatures in ccRCC development and progression were discussed and confirmed by the existed literature. The biological roles of the identified miRNA signatures were examined using enrichment analyses.

Future works: we will attempt to identify more robust features and classification models and apply different balanced data algorithms to improve the efficiency of the classification performance of early and late stage other than LR, Naive Bayes, KNN, and SVM classifiers.

## Conclusions

In summary, we have attempted to identify potential miRNA signatures for stratification of risk using integrated statistical approach. An optimal four-miRNA based prognostic model with the smallest AIC criterion was identified and the four-miRNA signature can effectively classify ccRCC patients into high and low risk groups. Moreover, using state-of-the-art ML algorithms, we identified six top ranked miRNA signatures that can classify early and late stages for patients with ccRCC. These six miRNA signature classified early stage and late stage with reasonable good classification performance. Finally, we explored the biological roles of the identified risk- and stage-associated miRNA signatures and these signatures involved important biological pathways. Overall, we expect that our findings provide promising insight to explore the role of miRNAs in ccRCC patients and could help risk and stage classifications.

## Methods

### Input data

The RNAseq expression profiles and their corresponding clinical data (including TNM stage, survival time, sex, age) for ccRCC were retrieved from the Firebrowse database (http://firebrowse.org/). The clinical information consists of 325 samples (254 tumors and 71 normal tissues), and clinical data consists of 131, 28, 48, and 45 samples of stage I, stage II, stage III and stage IV respectively. Finally, we considered both early stage (stage I and stage II, n = 159) and late stage (stage III and stage IV, n = 93) samples of patients for classification purpose.

### Preprocessing and identification of differentially expressed miRNAs

Normalization of the miRNA profiles were carried out by using the 'edgeR' package [19]. Differential analysis of miRNAs was conducted by utilizing the 'LIMMA' package in R [19] and miRNAs that satisfy the criterion of the absolute value of log2fold change > 1 and Benjamin-Hochberg (BH) adjusted *P*-value < 0.05 were considered as differentially expressed miRNAs (DEMs).

### Data standardization for ML modeling

In this calculation, z-scaling was used to normalized the count per million (CPM) of miRNA expression values, which is defined by the following equation.

1 $$\begin{aligned} x = & log_{2} (CPM + 1), \\ z = & \frac{{x - \overline{x}}}{\sigma }. \\ \end{aligned}$$

where x is the expression value of miRNA, $\overline{x}$ is the mean expression values of miRNA of the samples and σ is the standard deviation of expression values of miRNA of the samples and  z  represents normalized miRNA expression that follow the normal distribution with zero mean and unit standard deviation.

### Identification of miRNA signature associated patient survival and construction of risk classification model

Feature selection (FS) is an important process to improve classification performance by avoiding irrelevant/noise features. To select survival-associated features (miRNAs), Cox model regression model was proposed. Cox regression is defined as:

2 $$h\left( {t,X_{i\beta } } \right) = h_{0} \left( t \right){\text{exp}}\left( {X_{i\beta } } \right)$$

Here, *i* denotes for ccRCC patients, and the Xs are the covariates (miRNAs). $X_{i\beta } = \beta_{1} x_{i1} + \cdots + \beta_{k} x_{ik}$, $h_{0} \left( t \right)$ is the baseline hazard function at time *t*, $\beta = (\beta_{1} , \beta_{2} , \ldots ,\beta_{k} )$ is the vector of regression coefficients, and $\beta$ denotes the k-dimension regression coefficient vector of covariates. However, when we deal with high-dimension data such as genomic data, number of penalized Cox models have been proposed (including: Lasso, Adaptive lasso and Elastic-net) to solve the overfitting problem [31–33]. For each model, the estimated $\hat{\beta }$ values are obtained by minimizing the negative log-likelihood function with different penalty functions as follows:

3 $${\hat{\upbeta }}_{{{\text{Lasso}}}} = {\text{arg}}\;{\text{min}}_{{\upbeta }} \left\{ { - \mathop \sum \limits_{{{\text{i}} = 1}}^{{\text{n}}}\updelta _{{\text{i}}} \left( {{\text{X}}_{{{\text{i}}\upbeta }} - \log \left( {\mathop \sum \limits_{{{\text{i}} = 1}}^{{\text{n}}} \exp \left( {{\text{X}}_{{\text{i}}}\upbeta } \right)} \right)} \right) + { }\uplambda \mathop \sum \limits_{{{\text{i}} = 1}}^{{\text{K}}} \left| {\upbeta _{{\text{i}}} } \right|} \right\}$$

4 $${\hat{\upbeta }}_{{{\text{Ad}}.\,{\text{lasso}}}} = {\text{arg min}}_{\upbeta } \left\{ { - \mathop \sum \limits_{{{\text{i}} = 1}}^{{\text{n}}}\updelta _{{\text{i}}} \left( {{\text{X}}_{{{\text{i}}\upbeta }} - \log \left( {\mathop \sum \limits_{{{\text{i}} = 1}}^{{\text{n}}} \exp \left( {{\text{X}}_{{\text{i}}}\upbeta } \right)} \right)} \right) + 2\uplambda \mathop \sum \limits_{{{\text{i}} = 1}}^{{\text{K}}} {\text{w}}_{{{\text{nj}}}} \left| {\upbeta _{{\text{i}}} } \right|} \right\}$$

5 $${\hat{\upbeta }}_{{{\text{Enet}}}} = {\text{arg min}}_{\upbeta } \left\{ { - \mathop \sum \limits_{{{\text{i}} = 1}}^{{\text{n}}} {\updelta }_{{\text{i}}} \left( {{\text{X}}_{{{\text{i}}\upbeta }} - \log \left( {\mathop \sum \limits_{{{\text{i}} = 1}}^{{\text{n}}} \exp \left( {{\text{X}}_{{\text{i}}}\upbeta } \right)} \right)} \right) + { }\uplambda \mathop \sum \limits_{{{\text{i}} = 1}}^{{\text{K}}} \left| {\upbeta _{{\text{i}}} } \right| + (1 - \upalpha )\mathop \sum \limits_{{{\text{i}} = 1}}^{{\text{K}}}\upbeta _{{\text{i}}}^{2} } \right\}$$

Here $\delta_{i}$ is an indicator for the uncensored observation, λ is called penalty and $w_{nj}$ = $\left| {\hat{\beta }_{nj} } \right|^{ - 1}$ is calculated from the initial estimator $\hat{\beta }_{n}$.

Lasso Cox and adaptive Lasso Cox were used to identify relevant miRNAs associated with survival time by the shrinkage of some of the irrelevant miRNAs regression coefficients to zero. Adaptive Lasso Cox imposes an adaptive weighted penalty term in comparison with the Lasso, model, which further reduce the number of less-relevant miRNAs in such a way that the resulting coefficient estimates are sparse. Elastic-net is suitable for screening relevant miRNAs when there is a multicollinearity problem in the genomic data [34]. We used (ten-fold) cross-validation of Lasso, Adaptive lasso and Elastic-net algorithms to obtain the predicted optimal λ value for each method. The optimal λ value that minimize the estimated mean-squared prediction error and this optimal λ was used to select candidate miRNAs. All these methods were implemented using the ‘glmnet’ [35] package in *R*. Then, to make use of the strength of each method, we proposed a combined feature selection approach in the study of miRNA-disease association. The union of candidate miRNAs identified by the three algorithms were used to identify the best miRNAs combination that could predict prognosis of ccRCC patients more effectively. More detailed description of selecting the best subset prognostic model is described below:

Let $l = 1 , 2, \ldots . , k$, where *k* is the total number of candidate miRNAs identified by Lasso, Adaptive lasso and Elastic-net algorithms.

- Construct all possible combinatorial subset model having $l$ miRNA candidates
- Compare all possible models with the Akaike information criterion (AIC)
- Select the best subset prognostic model, having the smallest AIC, with $l$ miRNAs using the ‘glmulti’ package [36] in *R*.

After identifying the best subset prognostic model, we developed risk score (a linear combination of best subset miRNA expression and coefficients of miRNAs obtained from multivariate analysis) to evaluate risk prediction performance. The risk score (RS) computed as follow,

6 $${\text{Risk}}\;{\text{Score}} = \mathop \sum \limits_{i = 1}^{k} x_{i} \beta_{i}$$

Then, RS was used to classified ccRCC patients into high risk and low risk group using median RS as cutoff. The time dependent receiver operating characteristics (ROC) was used to estimate the survival time difference between high risk and low risk group using the ‘survivalROC’ package in R.

### Identification of miRNA signature associated with tumor stages for early and late tumor stage classification

To select best subset features (miRNAs) that associated with tumor stages, we proposed to use hybrid feature selection methods. Feature selection methods were conducted based on ten-fold cross-validation. The four popular ML algorithms proposed in this study include: LR, RF, SVMR and avNNet, and top ranked miRNAs based on their importance as the best features were identified from high-dimensional data. We utilized the “caret” package in *R* that consists of several complex ML algorithms for classification and prediction problems.

The processing of feature selection using four ML algorithms are briefly discussed as follows.

Ensemble logistic regression (LR) model is a ML model used as a classification model in feature selection to identify features that can distinguish binary samples of patients. Let ${\varvec{x}} \in {\varvec{R}}^{k}$ denotes an observation consists of $k$ feature miRNA values and let $y \in \left\{ { - 1, + 1} \right\}$ represents the corresponding binary outcome; such as, early and late stage. A LR model is a condition probability distribution (CPD) of the class level $y$ given the feature (miRNAs) vector ${\varvec{x}}$ is defined by

7 $$p\left( {y/{\varvec{x}}} \right) = \frac{1}{{1 + {\text{exp}}\left( { - y\left( {w^{T}x + v} \right)} \right)}}$$

where $\user2{w } \in R^{k }$ is the weight vector and $v \in {\varvec{R}}$ are parameters of LR. Ensemble LR for relevant feature selection is stable with respect to variation of the learning samples, since it uses t-test to rank features, which does not consider dependence between features. LR transfers the strongly correlated features to the LR gradient decent optimization algorithm to increase classification performance [37].

The random forest (RF) algorithm is a non-linear regression model, which has been used in several regression and classification problems in a variety of computational studies. RF learning is flexible algorithm that minimizes prediction errors and screen the most important features related to class level when feature size is large. It consists of constructing trees by splitting random sample of *r* features from a set of *n* features using bootstrapped training data.

8 $$IG\left( n \right) = 1 - \mathop \sum \limits_{i = 1}^{j} \left( {ki} \right)^{2}$$

where *IG* denotes impurity of a node n, $ki$ denotes the features (miRNAs), and *j* is the fraction of each $ki$ feature (miRNAs) [38].

Support vector machine (SVM) algorithm has been applied in many biological problems, such as biomarkers selection and classification problems [16, 39]. SVM is non-probabilistic classifier which maximize the margin of the decision boundary to classify two classes using support vectors to achieve the best classification. The general formulation of the SVM  classifier is defined as

9 $$minimize \frac{1}{2}w^{2} + \varphi \mathop \sum \limits_{i = 1}^{n} x_{i}$$

Here, *w* denotes the normal vector of the hyperplane, $\varphi$ is the classifier parameter, $x_{i}$ are the variables and *n* is the number of vectors in the training dataset.

The avNNet model is a type of neural network (NN), that learns nearly infinite number of mapping functions and works like natural human neurons. The inputs (features) connect to class label (staging), the connections are called edges. The input feature connects either forward/backward propagation hidden nodes to compute neurons. The number of hidden layers determines the depth of NN. A feedforward NN having more than one hidden layer is called deep network [40, 41]. The NN is commonly applied to the discovery of biomarkers in cancer studies [42].

10 $$\beta_{j}^{r} = \vartheta \left( {\mathop \sum \limits_{K} w_{jk}^{r} \beta_{k}^{r - 1} + \alpha_{j}^{r} } \right)$$

where $\beta_{j}^{r}$ represents the *j*th neuron in the *r*th layer is associated with activation in the (*r* − 1)th layer. The $\vartheta$ is a vectorising function parameter. The $w_{jk}^{r}$ denotes a *r*th weight matrix for each layer *r* of the *j*th row and *k*th column, and $\alpha_{j}^{r}$ denotes the *j* bias for each layer *r*.

### Stage classification methods

SVMR, NB, avNNet, KNN and LR were utilized for building predictive and classification models. Each model was constructed by ten-fold cross validation to avoid over/under fitting. The cost function was optimized [100–1000 iterations with 100 steps per iteration] to attain accurate classification.

### Data balancing

The proportion of early stage patients is approximately twice of late stage patients; this creates the data imbalance problem which leads to biased prediction. Therefore, we performed the data balancing procedure by using the Synthetic Minority Oversampling Technique (SMOTE) algorithm [43] (included in the DMwR package). The SMOTE is a common method to solve data imbalance problem more effectively, prior to applying the ML classifier [42]. The dataset was divided randomly into training set (80%) and the remaining 20% as independent test set. The training set was employed to train ML algorithms in classifying early and late stage of patients based on ten-fold cross validation. The test set was used as independent test set and used to assess the classification performance of five ML methods.

### Classification performance evaluation metrics

To evaluate the performance of the classification models, we used the following measures: specificity, sensitivity, accuracy (ACC), precision, and Matthews correlation coefficient (MCC). The mathematical formulas of the measures are given below:

11 $$Sensitivity = \frac{TP}{{TP + FN}}$$

12 $$Specificity = \frac{TN}{{TN + FP}}$$

13 $$ACC = \frac{TP + TN}{{TP + TN + FP + FN}}$$

14 $$Precision = \frac{TP}{{TP + FP}}$$

15 $${\text{MCC}} = \frac{TP \times TN - FP \times FN}{{\sqrt {(TP + FP)(TP + FN)(TN + FP)(TN + FN)} }}$$

where TP, TN, FP, and FN denote true positive, true negative, false positive and false negative respectively.

### Functional enrichment analysis

We assessed the biological relevance of the identified prognostic and diagnostic miRNA signatures using the DIANA-mirPath [44].

## Supplementary Information

**Additional file 1**. Lists of supplementary materials, including workflow, differentially expressed miRNAs, KM plots of ccRCC patients, Cox regression results, and the results of enrichment analysis of the miRNA signatures.

## Data Availability

The datasets analyzed during the current study are available in the Firebrowse database, https://gdac.broadinstitute.org/.

## Abbreviations

RCC: Renal cell carcinoma
ccRCC: Clear cell renal cell carcinoma
ML: Machine learning
TCGA: The Cancer Genome Atlas
SMOTE: Synthetic Minority Oversampling Technique
LASSO: Least absolute shrinkage and selection operator
HR: Hazard ratio
SVM: Support Vector Machine
SVMR: Support Vector Machine with radial kernel
MRMR: Maximum Relevance Minimum Redundancy
LR: Logistic regression
KNN: K-nearest neighbors algorithm
AVNNET: Averaging Neural network
GO: Gene Ontology
KEGG: Kyoto Encyclopedia of Genes and Genomes
