# Supplementary material for: A novel miRNA-based classification model of risks and stages for clear cell renal cell carcinoma patients
Source: BMC Bioinformatics. 2021 May 25;22(Suppl 10):270. doi: 10.1186/s12859-021-04189-2 (PMC8323484; doi:10.1186/s12859-021-04189-2)
Supplement: Supplementary file 1 — Additional file 1. Lists of supplementary materials, including workflow, differentially expressed miRNAs, KM plots of ccRCC patients, Cox regression results, and the results of enrichment analysis of the miRNA signatures. [file 12859_2021_4189_MOESM1_ESM.docx]

**A novel miRNA-based classification model of risks and stages for clear cell renal cell carcinoma patients**

Eskezeia Y. Dessie ^1, 4^, Jeffrey J.P. Tsai^1^, Jan-Gowth Chang^2 *^, Ka-Lok Ng^1, 3, 4 †^

^1^Department of Bioinformatics and Medical Engineering, Asia University, Taiwan

^2^ Department of Laboratory Medicine, China Medical University, Taiwan

^3^ Department of Medical Research, China Medical University Hospital, China Medical University, Taiwan

^4^ Center for Artificial Intelligence and Precision Medicine Research, Asia University, Taiwan

*^†^ To whom correspondence should be addressed.

**Contact:** ^†^ Ka-Lok Ng:[ppiddi@gmail.com](mailto:ppiddi@gmail.com), * Jan-Gowth Chang: d6781@mail.cmuh.org.tw


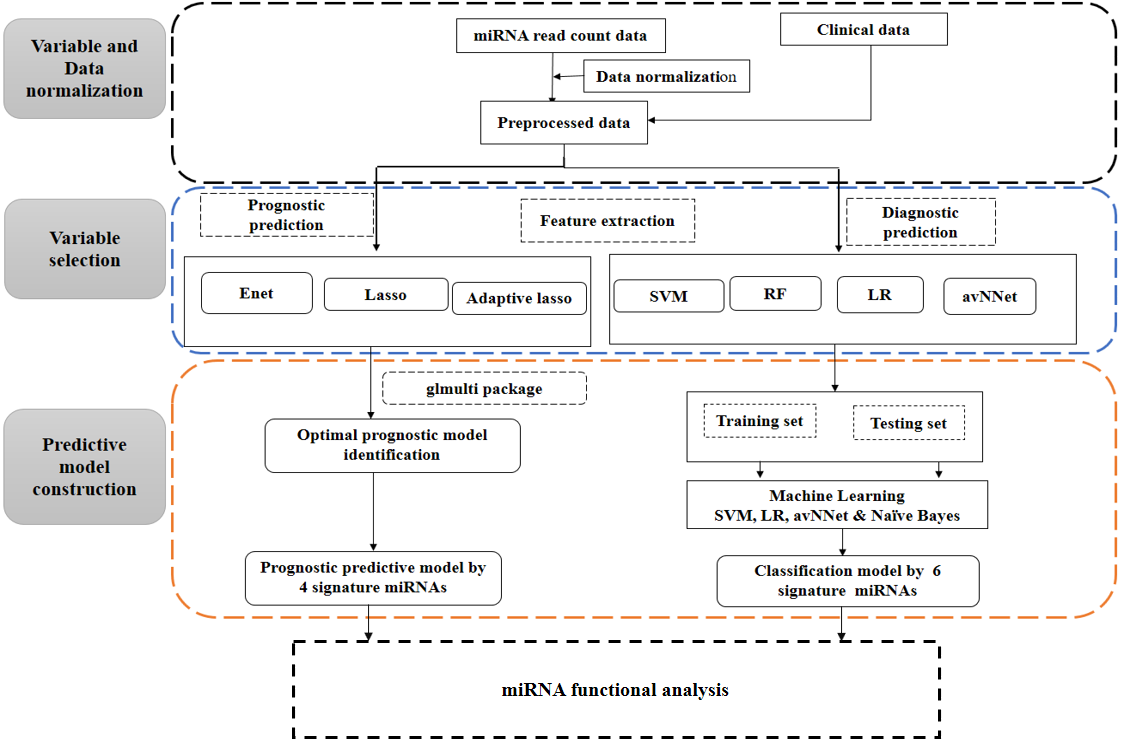


**Additional file 1: Figure** S1. Work flow of our study

The overall methodology consists of the following steps: i) data processing, ii) prognostic and diagnostic related features(miRNAs) selection iii) prognostic and diagnostic model construction iv) Functional analysis of identified miRNA signature.


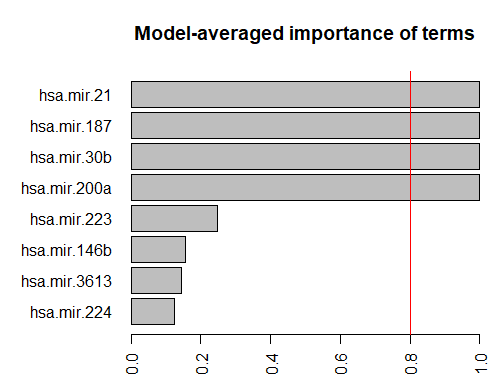


**Additional file 1: Figure S2**: Top eight features identified by best subset analysis (BSR) using multivariate Cox regression. The miRNAs that were selected by the optimal model were marked with a red dot. The red line shows the 80% threshold for importance.

**Additional file 1: Table S1**: Summary of differentially expressed miRNAs (DEMs) in ccRCC samples compared with normal tissue samples based on TCGA cohorts.

| **DEMs** | **DEMs (\|log2 fold change \|** $\boldsymbol{>1}$ **and adjusted P-value < 0.05** |
| --- | --- |
| Downregulated miRNAs  (N = 80) | miR-141, miR-200c, miR-891a, miR-508, miR-129-1, miR-184, miR-514-3, miR-514-2,  miR-514-1, miR-129-2, miR-206, miR-187, miR-362, miR-506, miR-934, miR-138-1,  miR-675, miR-203, miR-9-2, miR-9-1, miR-509-3, miR-1251, miR-135a-1, miR-429,  miR-509-1, miR-138-2, miR-509-2, miR-363, miR-514b, miR-149, miR-483, miR-188,  miR-31, miR-200b, miR-204, miR-216b, miR-199b, miR-199a-1, miR-199a-2, miR-3065, miR-136, miR-1269, miR-335, miR-874, miR-33a, miR-577, miR-214, miR-375, miR-500a, miR-200a, miR-20b, miR-26a-1, miR-154, miR-217, miR-1-2, miR-501, miR-337,  miR-135a-2, miR-539, miR-127, miR-183, miR-660, miR-30b, miR-205, miR-10a, miR-532, miR-323b, miR-369, miR-323, miR-376c, miR-133a-1, miR-500b, miR-411, miR-507,  miR-30c-2, miR-502, miR-135b, miR-493, miR-513c, miR-433 |
| Upregulated miRNAs  (N = 44) | miR-122, miR-210, miR-155, miR-885, miR-21, miR-592, miR-224, miR-584, miR-452,  miR-142, miR-629, miR-1270-1, miR-2355, miR-3613, miR-1270-2, miR-181b-1, miR-144, miR-215, miR-15a, miR-342, miR-146b, miR-93, miR-106b, miR-330, miR-599, miR-193a, miR-3941, miR-16-2, miR-223, miR-3130-1, miR-486, miR-25, miR-760, miR-374a,  miR-181a-1, miR-451, miR-34a, miR-625, miR-2277, miR-3615, miR-146a, miR-185,  miR-340, miR-576 |


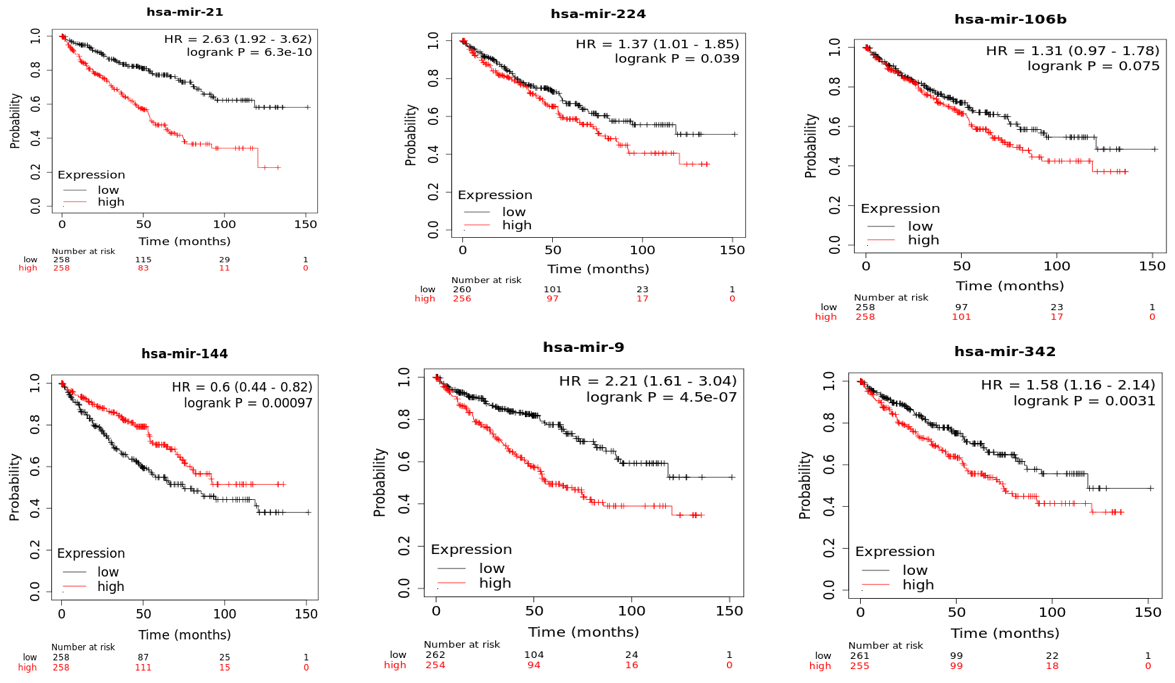


**Additional file 1: Figure S3**. Kaplan-Meier plotter show the survival time difference between high expression and low expression groups of stage related miRNAs in ccRCC patients.

**Additional file 1: Table S2:** Result of multivariate Cox analysis of four-miRNA signatures

| **Model** | **Multivariate Model** | | |
| --- | --- | --- | --- |
|  | **P-value** | **Coefficient** | **HR(95 % CI)** |
| miR-21 | $1.05\times{10}^{-09}$ | 1.485 | 4.416(2.740-7.115) |
| miR-200a | $8.44\times{10}^{-04}$ | -0.320 | 0.726(0.602-0.876) |
| miR-187 | $9.59\times{10}^{-04}$ | 0.485 | 1.624(1.218-2.165) |
| miR-30b | $1.18\times{10}^{-03}$ | 0.525 | 1.691(1.231-2.322) |

| **RF** | | **avNNet** | | **LR** | | **SVMR** | |
| --- | --- | --- | --- | --- | --- | --- | --- |
| **Feature** | **RI** | **Feature** | **RI** | **Feature** | **RI** | **Feature** | **RI** |
| miR-9-1 | 100 | miR-106b | 100 | miR-506 | 100 | miR-9-1 | 100 |
| miR-144 | 96.16 | miR-9-1 | 96.4 | miR-21 | 70.91 | miR-9-2 | 90.14 |
| miR-1269 | 93.87 | miR-142 | 92.88 | miR-106b | 55.68 | miR-144 | 89.19 |
| miR-486 | 87.76 | miR-9-2 | 89.23 | miR-675 | 52.24 | miR-486 | 89.1 |
| miR-9-2 | 80.74 | miR-486 | 89.14 | miR-184 | 50.03 | miR-106b | 84.52 |
| miR-193a | 80.21 | miR-144 | 86.02 | miR-509-1 | 34.32 | miR-21 | 84.07 |
| miR-452 | 78.7 | miR-21 | 83.89 | miR-31 | 32.91 | miR-342 | 79.28 |
| miR-106b | 77.72 | miR-1270-2 | 79.44 | miR-592 | 32.75 | miR-1269 | 78.64 |
| miR-21 | 73.31 | miR-342 | 79.24 | miR-337 | 32.35 | miR-193a | 74.52 |
| miR-183 | 70.77 | miR-1270-1 | 77.59 | miR-342 | 31.69 | miR-215 | 71.81 |
| miR-20b | 70.63 | miR-3613 | 76.2 | miR-224 | 30.13 | miR-149 | 71.09 |
| miR-30b | 70.5 | miR-183 | 73.38 | miR-199b | 29.54 | miR-224 | 68.19 |
| miR-330 | 69 | miR-155 | 72.08 | miR-30b | 29.32 | miR-142 | 67.29 |
| miR-224 | 67.19 | miR-224 | 71.56 | miR-216b | 28.64 | miR-3613 | 64.93 |
| miR-874 | 67 | miR-193a | 70 | miR-144 | 28.33 | miR-183 | 63.89 |
| miR-342 | 66.35 | miR-149 | 69.21 | miR-9-1 | 28.01 | miR-146b | 62.4 |
| miR-10a | 65.54 | miR-1269 | 67.56 | miR-129-1 | 27.26 | miR-592 | 55.29 |
| miR-149 | 63.25 | miR-509-2 | 66.63 | miR-15a | 25.73 | miR-20b | 54.43 |
| miR-93 | 62.75 | miR-10a | 66.04 | miR-874 | 25.14 | miR-199b | 53.03 |
| miR-25 | 62.75 | miR-215 | 65.44 | miR-26a-1 | 24.42 | miR-506 | 51.76 |

**Additional file 1: Table S3**: Twenty informative features identified by four machine learning algorithms. RI, relative importance

**Additional file 1: Table S4.** The performance of machine learning based model constructed by identified six miRNA signatures on the training set and testing set without data balancing.

| Algorithms | Methods | Performance Measures | | | | | |
| --- | --- | --- | --- | --- | --- | --- | --- |
|  |  | ACC | Sensitivity | specificity | MCC | F-score | Precision |
| SVMR | 10-fold | **0.781** | 0.875 | 0.758 | 0.521 | 0.609 | 0.467 |
|  | Testing | 0.674 | 0.222 | 0.936 | 0.232 | 0.333 | 0.667 |
| LR | 10-fold | 0.695 | 0.614 | 0.726 | 0.317 | 0.530 | 0.467 |
|  | Testing | 0.612 | 0.222 | 0.839 | 0.080 | 0.296 | 0.444 |
| Naïve Bayes | 10-fold | 0.778 | 0.75 | 0.790 | 0.511 | 0.667 | 0.600 |
|  | Testing | 0.775 | 0.778 | 0.742 | 0.503 | 0.700 | 0.636 |
| avNNet | 10-fold | 0.773 | 0.738 | 0.789 | 0.436 | 0.662 | 0.661 |
|  | Testing | 0.694 | 0.444 | 0.839 | 0.309 | 0.516 | 0.615 |
| KNN | 10-fold | 0.724 | 0.694 | 0.734 | 0.380 | 0.548 | 0.453 |
|  | Testing | 0.738 | 0.500 | 0.871 | 0.405 | 0.581 | 0.692 |

**Additional file 1: Table S5.** Upregulated miRNA signatures associated with risk and tumor stage: enriched KEGG pathways.

| **KEGG pathway** | **p-value** | **#genes** | **#miRNAs** |
| --- | --- | --- | --- |
| Fatty acid biosynthesis | 0 | 3 | 1 |
| Adherens junction | 1.76E-07 | 20 | 2 |
| Fatty acid metabolism | 5.63E-07 | 7 | 1 |
| Lysine degradation | 7.62E-05 | 13 | 2 |
| Oocyte meiosis | 0.00015 | 25 | 1 |
| Glycosphingolipid biosynthesis - ganglio series | 0.00017 | 1 | 1 |
| Mucin type O-Glycan biosynthesis | 0.00039 | 6 | 1 |
| Pathways in cancer | 0.00111 | 65 | 2 |
| Viral carcinogenesis | 0.00125 | 31 | 2 |
| Ubiquitin mediated proteolysis | 0.00253 | 30 | 1 |
| p53 signaling pathway | 0.00839 | 15 | 1 |
| mRNA surveillance pathway | 0.02678 | 25 | 2 |
| Bacterial invasion of epithelial cells | 0.03562 | 19 | 2 |
| Glycosaminoglycan biosynthesis - heparan sulfate / heparin | 0.03861 | 4 | 1 |
| RNA degradation | 0.04562 | 4 | 1 |

**Additional file 1: Table S6**. Downregulated miRNAs signatures associated with risk and tumor stage: enriched KEGG pathway

| KEGG pathway | p-value | #genes | #miRNAs |
| --- | --- | --- | --- |
| Adherens junction | 0.00012871 | 12 | 1 |
| Lysine degradation | 0.000273755 | 7 | 1 |
| ECM-receptor interaction | 0.002364666 | 8 | 1 |
| Steroid biosynthesis | 0.004888375 | 3 | 1 |
| Viral carcinogenesis | 0.01095091 | 17 | 1 |
| Proteoglycans in cancer | 0.03059655 | 18 | 1 |
| Apoptosis | 0.0323124 | 10 | 1 |
| Transcriptional misregulation in cancer | 0.03646795 | 20 | 1 |
| mRNA surveillance pathway | 0.03855594 | 13 | 1 |
| RNA transport | 0.03929878 | 21 | 1 |
| NF-kappa B signaling pathway | 0.04282901 | 9 | 1 |
| Bacterial invasion of epithelial cells | 0.04282901 | 11 | 1 |
| Hepatitis B | 0.04282901 | 12 | 1 |

**Additional file 1: Table S7**. Upregulated miRNAs signatures associated with risk and tumor stage: enriched GO annotations

| GO Category | p-value | #genes | #miRNAs |
| --- | --- | --- | --- |
| mitotic cell cycle | 0 | 98 | 3 |
| protein binding transcription factor activity | 0 | 108 | 3 |
| nucleic acid binding transcription factor activity | 0 | 182 | 3 |
| RNA binding | 0 | 356 | 3 |
| cellular component | 0 | 2283 | 3 |
| nucleoplasm | 0 | 269 | 3 |
| cytosol | 0 | 494 | 3 |
| cellular protein modification process | 0 | 440 | 3 |
| biological process | 0 | 2228 | 3 |
| catabolic process | 0 | 340 | 3 |
| biosynthetic process | 0 | 724 | 3 |
| viral process | 0 | 121 | 3 |
| enzyme binding | 0 | 258 | 3 |
| cellular component assembly | 0 | 227 | 3 |
| ion binding | 0 | 952 | 3 |
| protein complex | 0 | 642 | 3 |
| cellular protein metabolic process | 0 | 97 | 3 |
| small molecule metabolic process | 0 | 389 | 3 |
| symbiosis, encompassing mutualism through parasitism | 0 | 133 | 3 |
| poly(A) RNA binding | 0 | 296 | 3 |
| membrane organization | 0 | 133 | 3 |
| macromolecular complex assembly | 0 | 161 | 3 |
| molecular function | 0 | 2333 | 4 |
| gene expression | 0 | 159 | 4 |
| cellular nitrogen compound metabolic process | 0 | 884 | 4 |
| nucleobase-containing compound catabolic process | 0 | 176 | 4 |
| organelle | 0 | 1736 | 4 |
| Fc-epsilon receptor signaling pathway | 1.11E-16 | 43 | 3 |
| response to stress | 3.38E-14 | 342 | 3 |
| cell death | 4.55E-13 | 160 | 3 |
| protein complex assembly | 1.86E-12 | 134 | 3 |
| transcription, DNA-templated | 3.32E-12 | 388 | 3 |
| neurotrophin TRK receptor signaling pathway | 6.38E-12 | 54 | 3 |
| cytoskeletal protein binding | 1.21E-11 | 100 | 2 |
| post-translational protein modification | 3.62E-11 | 42 | 3 |
| blood coagulation | 4.22E-11 | 86 | 3 |
| mRNA metabolic process | 9.23E-10 | 43 | 3 |
| enzyme regulator activity | 1.85E-08 | 124 | 2 |
| RNA metabolic process | 5.34E-08 | 43 | 2 |
| epidermal growth factor receptor signaling pathway | 7.37E-07 | 47 | 3 |
| in utero embryonic development | 3.40E-06 | 68 | 3 |
| DNA metabolic process | 4.05E-06 | 110 | 2 |
| RNA splicing | 5.26E-06 | 47 | 2 |
| mitotic nuclear envelope disassembly | 5.76E-06 | 12 | 2 |
| platelet activation | 8.85E-06 | 41 | 3 |
| vesicle-mediated transport | 9.25E-06 | 163 | 3 |
| transcription initiation from RNA polymerase II promoter | 1.27E-05 | 30 | 1 |
| toll-like receptor 10 signaling pathway | 1.42E-05 | 17 | 2 |
| chromatin organization | 2.30E-05 | 26 | 2 |
| TRIF-dependent toll-like receptor signaling pathway | 3.30E-05 | 18 | 2 |

**Additional file 1: Table S8**. Downregulated miRNA signatures associated with risk and tumor stage: enriched GO annotations

| GO Category | p-value | #genes | #miRNAs |
| --- | --- | --- | --- |
| nucleobase-containing compound catabolic process | 0 | 146 | 2 |
| macromolecular complex assembly | 0 | 136 | 2 |
| mitotic cell cycle | 0 | 98 | 3 |
| cellular protein modification process | 0 | 440 | 3 |
| biological process | 0 | 2228 | 3 |
| catabolic process | 0 | 340 | 3 |
| biosynthetic process | 0 | 724 | 3 |
| gene expression | 0 | 155 | 3 |
| viral process | 0 | 121 | 3 |
| cellular component assembly | 0 | 227 | 3 |
| cellular nitrogen compound metabolic process | 0 | 872 | 3 |
| cellular protein metabolic process | 0 | 97 | 3 |
| small molecule metabolic process | 0 | 389 | 3 |
| symbiosis, encompassing mutualism through parasitism | 0 | 133 | 3 |
| membrane organization | 0 | 133 | 3 |
| Fc-epsilon receptor signaling pathway | 6.66E-16 | 36 | 2 |
| response to stress | 1.02E-13 | 152 | 1 |
| cell death | 1.44E-12 | 132 | 2 |
| protein complex assembly | 5.23E-12 | 117 | 2 |
| transcription, DNA-templated | 1.13E-11 | 298 | 2 |
| neurotrophin TRK receptor signaling pathway | 2.24E-11 | 19 | 1 |
| post-translational protein modification | 1.13E-10 | 25 | 1 |
| blood coagulation | 1.25E-10 | 44 | 1 |
| mRNA metabolic process | 2.02E-09 | 26 | 1 |
| RNA metabolic process | 1.01E-07 | 27 | 1 |
| transcription initiation from RNA polymerase II promoter | 2.63E-05 | 30 | 1 |
| toll-like receptor 10 signaling pathway | 3.04E-05 | 11 | 1 |
| chromatin organization | 4.42E-05 | 18 | 1 |
| extracellular matrix disassembly | 6.81E-05 | 17 | 1 |
| viral life cycle | 0.000116 | 16 | 1 |
